# Hypothesis: Chemical activity regulates and coordinates the processes maintaining glycerophospholipid homeostasis in mammalian cells

**DOI:** 10.1096/fba.2019-00058

**Published:** 2020-01-27

**Authors:** Pentti Somerharju, Jorma A. Virtanen, Martin Hermansson

**Affiliations:** ^1^ Medicum Faculty of Medicine University of Helsinki Helsinki Finland; ^2^ Wihuri Research Institute Helsinki Finland

**Keywords:** coordination, homeostasis, maintenance, metabolism, set point

## Abstract

Mammalian cells maintain the complex glycerophospholipid (GPL) class compositions of their various membranes within close limits because this is essential to their well‐being or viability. Surprisingly, however, it is still not understood how those compositions are maintained except that GPL synthesis and degradation are closely coordinated. Here, we hypothesize that abrupt changes in the chemical activity of the individual GPL classes coordinate synthesis and degradation as well other the homeostatic processes. We have previously proposed that only a limited number of “allowed” or “optimal” GPL class compositions exist in cellular membranes because those compositions are energetically more favorable than others, that is, they represent local free energy minima (Somerharju et al 2009, Biochim. Biophys. Acta 1788, 12‐23). This model, however, could not satisfactorily explain how the “optimal” compositions are sensed by the key homeostatic enzymes, that is, rate‐limiting synthetizing enzymes and homeostatic phospholipases. We now hypothesize that when the mole fraction of a GPL class exceeds an optimal value, its chemical activity abruptly increases which (a) increases its propensity to efflux from the membrane thus making it susceptible for hydrolysis by homeostatic phospholipases; (b) increases its potency to inhibit its own biosynthesis via a feedback mechanism; (c) enhances its conversion to another glycerophospholipid class via a novel process termed “head group remodeling” or (d) enhances its translocation to other subcellular membranes. In summary, abrupt change in the chemical activity of the individual GPL classes is proposed to regulate and coordinate those four processes maintaining GPL class homeostasis in mammalian cells.

**Abbreviations**
CCT: CTP:phosphocholine cytidylyltransferase
CL: cardiolipin
DAG: diacylglycerol
GPL: glycerophospholipid
PA: phosphatidic acid
PC: phosphatidylcholine
PE: phosphatidylethanolamine
PG: phosphatidylglycerol
PI: phosphatidylinositol
PLA: phospholipase A
PLC: phospholipase C
PS: phosphatidylserine
SL: superlattice
TAG: triacylglycerol

## 1 INTRODUCTION

**Figure 1:**
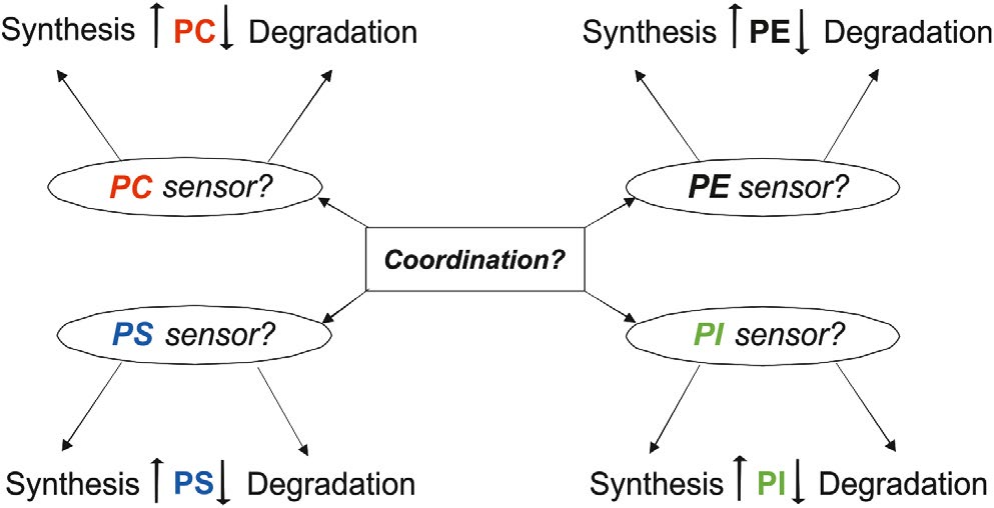
Complexity of regulation of the glycerophospholipid (GPL) compositions of mammalian membranes. This scheme emphasizes the complexity of regulation of the GPL compositions of membranes consisting of many different lipid classes. All GPL classes present in mammalian cells are not shown here for simplicity

Glycerophospholipids (GPLs) form the backbone of all membranes in mammalian cells. The major GPL classes are phosphatidylcholine (PC), ‐ethanolamine (PE), ‐inositol (PI), ‐serine (PS), ‐glycerol (PG), phosphatidic acid (PA) and cardiolipin (CL) and the relative concentrations (mole fractions) of these GPLs are kept within close limits in mammalian cells and tissues^1^ apparently because deviations from the “optimal” composition can have dire consequences.^2^, ^3^, ^4^, ^5^, ^6^ Remarkably, however, despite the vital importance of GPL homeostasis, the mechanisms underlying this crucial phenomenon is poorly understood, except for that biosynthesis and degradation are tightly coordinated. Such coordination is demonstrated, for example, by that when the synthesis of PC was increased several‐fold, its concentration remained essentially unchanged due to increased degradation.^7^, ^8^, ^9^, ^10^, ^11^ Parallel evidence has been obtained for PE and PS.^7^, ^9^, ^11^, ^12^ Conversely, when the synthesis of PC, PE or PS was inhibited, their turnover decreased correspondingly.^13^, ^14^, ^15^, ^16^, ^17^ However, there it is no information on how the synthesis and degradation are coordinated, which must be a challenging task due to the presence of many GPL classes in the same membrane (Figure 1). The key challenge derives from the fact that when the mole fraction (relative concentration) of a single GPL class changes, the mole fractions of all other GPL classes are simultaneously altered. Accordingly, the mechanisms controlling the mole fractions of the individual GPL classes must be acutely and accurately coordinated to maintain homeostasis. As far as we are aware, no model or theory on how the coordination is accomplished has been put forward.

Here, we present a hypothesis proposing that the abrupt, composition‐dependent changes in the chemical activity of the individual GPL classes regulate and coordinate their synthesis and degradation thus maintaining GPL homeostasis in mammalian cells. This hypothesis, inspired by our recent findings on the processes involved in GPL homeostasis, represents a major extension of the previously proposed Superlattice model.

### 1.1 Superlattice model and its shortcomings

We have previously shown that the GPL compositions of the inner and outer leaflets of mammalian erythrocyte and platelet membranes are remarkably similar to compositions predicted by the so‐called Superlattice (SL) Model, which proposes that there is a limited number of “allowed” GPL mole fractions.^18^, ^19^ Accordingly, the relative concentrations of the different phospholipid classes tend to settle in “allowed” values because that provides the optimal interaction between the proximal molecules, that is, a free energy minimum. The model could not, however, adequately explain how the “allowed” compositions are maintained in the membranes of nucleated cells in which the GPLs are continuously synthetized and degraded.

Regarding synthesis, the SL‐model proposed that when the mole fraction of a particular GPL reaches a value allowed by the model, membrane lateral order increases abruptly which leads to aggregation of the respective synthetizing enzyme thus inactivating it.^19^ Correspondingly, when the mole fraction of the particular GPL class falls below its critical value, the superlattice would collapse and, consequently, membrane order would drop abruptly thus reactivating of the enzyme synthetizing the particular GPL. As far as we are aware, such regulatory mechanism is not supported by the data published thus far and seems thus unlikely.

Regarding degradation, the SL‐model proposed that when the mole fraction of a particular GPL exceeds a critical value, segregated lateral domains would appear and then homeostatic phospholipases, activated by poorly packed domain boundaries, would hydrolyze the GPL molecules in excess. Once the GPL in excess had been degraded, the segregated domains and the boundaries would disappear thus rendering the phospholipases inactive. A serious shortcoming of this model is that it could not explain why only the molecules in excess would be degraded by homeostatic phospholipases? In conclusion, it remained speculative how the synthesis and degradation of GPLs are regulated and coordinated so that homeostasis is maintained in growing cells in which GPLs are continuously synthetized and degraded.

**Figure 2:**
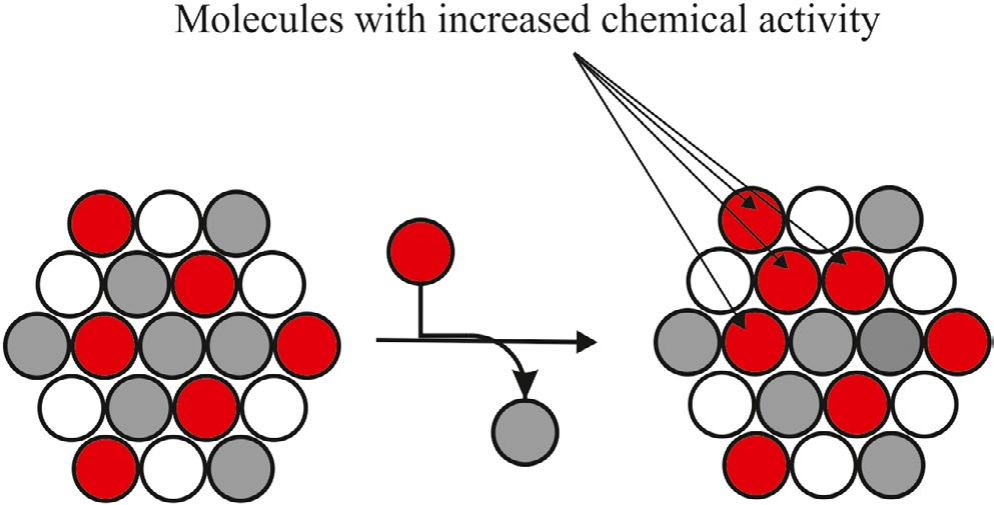
Deviation from an optimal composition brings about several glycerophospholipid (GPL) molecules with an increased chemical activity. On the left: The GPL class composition is optimal as proposed previously for the erythrocyte membrane inner leaflet where PE (gray) is ~44 mol%, the choline lipids (white) are ~22 mol% and the negatively charged GPLs (red) are ~33 mol%.^18^ Note that at this composition there are no proximal (strongly repelling) negatively charged GPLs. On the right: If a (zwitterionic) GPL molecule is replaced by a negatively charged one, the chemical activity of three or four negatively charged GPL molecules is greatly increased due to electrostatic repulsion between the proximal negatively charged GPLs. If the mole fraction of an zwitterionic GPL increases above its optimal value (not shown here), its chemical activity is predicted to increase due to weakened van der Waals or hydrogen bonding interactions with its neighbors, or steric strain

**Figure 3:**
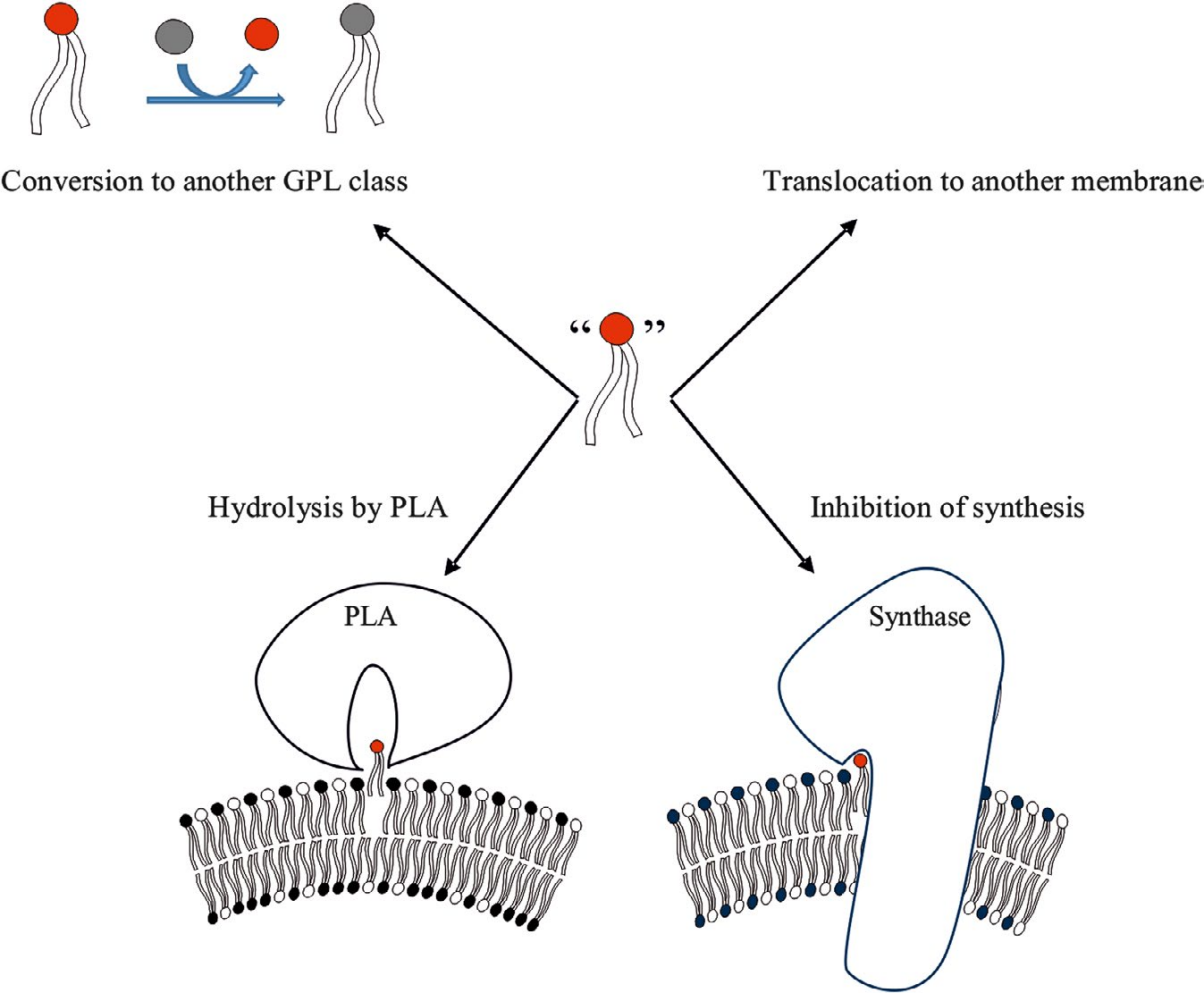
Multiple homeostatic events can be driven by increased chemical activity of the glycerophospholipids (GPLs) present in excess. As discussed in the text, the GPL molecules present in excess (red) has increased chemical activity which is predicted to (a) increase its hydrolysis by a phospholipase A; (b) inhibit its own biosynthesis; (c) enhance its conversion to another GPL with a different head group (=head group remodeling), or (d) enhance its translocation to another membrane. All these events are may occur simultaneously to maintain GPL class homeostasis in mammalian cells

Due to the shortcomings indicated above as well as recent novel data of the synthesis and degradation of GPLs^20^, ^21^, ^22^, ^23^ we hypothesize here that the *chemical activity* of the different GPLs regulate and coordinate their metabolism thus maintaining the GPL homeostasis. We propose (a) that when the mole fraction of one GPL class deviates from an optimal value, its chemical activity changes abruptly due to weakened interactions with the proximal molecules (Figure 2) and (b) such abrupt changes in the chemical activity of the different GPLs regulate and coordinate multiple homeostatic process including synthesis, degradation, interconversion (head group remodeling), and interorganelle translocation of GPLs (Figure 3). Below we will discuss in more detail the evidence suggesting that chemical activity could indeed regulate each of these processes.

### 1.2 Chemical activity regulates GPL biosynthesis

Excluding PS and PC, it is poorly established what regulates the biosynthesis of GPLs in mammalian cells. Kuge and coworkers have demonstrated that PS strongly inhibits its own synthesis in CHO cells and that this inhibition is most probably mediated by the interaction of PS with a specific arginine in PS synthase 1 or 2 (reviewed in ref. [24]). In the synthesis of PC the rate limiting, and thus the regulatory step, is the binding of CTP:phosphocholine cytidylyltransferase (CCT) to the ER or nuclear membrane.^25^ The binding is inhibited by lyso‐PC and stimulated by PE, diacylglycerol (DAG) and negatively charged lipids, and it has been proposed that the ratio of those lipids differently modulate the membrane packing (or curvature elastic stress) or charge of the ER membrane thus regulating CCT binding to the membrane.^26^, ^27^ Early studies have also indicated that, beside PS and PC, the synthesis of PI may also be regulated by a feed‐back mechanism in rat pituitary cells,^28^ but the details of this process remain unclear. Recently, we have shown that loading of any common GPL to HeLa, BHK‐21, or CHO cells strongly inhibited the synthesis of the corresponding GPL class.^22^, ^23^ The GPL molecules in excess in a membrane should have an increased chemical activity, which should promote their binding to the active or a regulatory site of the synthetizing enzyme thus inhibiting its activity. Alternatively, the molecules in excess could, for example, in case of CTT, inhibit membrane association and thus the activity of the synthetizing enzyme. In conclusion, chemical activity of GPLs is proposed to be the factor regulating the rate‐limiting enzymes of biosynthesis via a feed‐back mechanism. Previously, chemical activity of cholesterol has been suggested to regulate its biosynthesis.^29^, ^30^

### 1.3 Increased chemical activity renders GPLS susceptible to hydrolysis by homeostatic phospholipases

There is good evidence that Ca^2+^‐independent PLAs (iPLAs alias PNPLAs) are the key players in homeostatic degradation of GPLs in mammalian cells^9^, ^10^, ^31^, ^32^, ^33^ as discussed in more detail elsewhere.^1^ Consistently, we have recently shown that PNPLA9, −6, and −4 catalyze homeostatic degradation of PC, PE, and PS in human cells.^21^ More importantly, we have provided strong evidence that the activity of PNPLA9 in vitro is proportional to the propensity of its GPL substrate to efflux from the membrane^20^ to the active site of PNPLA9, predicted to reside well above the membrane surface.^34^ Since the efflux propensity of a GPL molecule should be proportional to its chemical activity, we propose that the rate of homeostatic degradation of GPLs correlates positively on their chemical activity.

### 1.4 Chemical activity drives GPL glass interconversion (head group remodeling)

We have recently found that exogenous PE, PS, PI, PG, and PA are rapidly and effectively converted to PC when loaded to HeLa cells.^22^, ^23^ Notably, blocking of fatty acyl‐CoA formation with Triacsin C had no effect on the conversion to PC thus excluding the possibility that deacylation of the GPL precursor, followed by incorporation of released fatty acids to PC *via* synthesis de novo is involved in the process. Extensive knock‐down studies indicated that different enzymes (including PLCs or similar enzymes) catalyze the initial, committed step of the interconversion or “head group remodeling”.^23^ Since loading of an exogenous GPL to the cells should greatly increase the chemical activity of the respective GPL class, it is most likely that chemical activity drives head group remodeling. A particular benefit of this novel homeostatic process is that it requires far less cellular energy than biosynthesis de novo*,* simply because the fatty acids need not to be activated to CoA derivatives.

### 1.5 Interorganelle translocation of GPLs, yet another process affected by chemical activity

As suggested earlier, when the molar fraction of a GPL class increases above an optimal value, its chemical activity and thus its propensity to efflux from a membrane should increase abruptly. It has been previously shown that the rate‐limiting step in spontaneous intermembrane translocation of a lipid is its efflux from the donor membrane.^35^, ^36^ While spontaneous intermembrane translocation of lipids is often considered negligible, this does not apply to all lipids as their hydrophobicity varies by orders of magnitude.^37^ Notably, efflux from the donor membrane seems to be the rate‐limiting step in protein‐mediated translocation processes as well.^38^, ^39^ It is also worthy to note that inter‐organelle translocation of a GPL is necessarily coupled to its biosynthesis,^40^ since the translocation affects the concentration of that GPL both in the donor and acceptors membranes and thus the efficiency of feedback inhibition of biosynthesis in either membrane. In conclusion, if the mole fraction of a GPL in a membrane increases, its chemical activity and, consequently, its intracellular translocation is also likely to increase as has been previously proposed for cholesterol.^29^, ^41^, ^42^

### 1.6 Other modes of regulation

Finally, we stress that beside the ones proposed here there are also other mechanisms that regulate GPL composition of mammalian cells, such as those depending on altered gene expression or translation. However, those mechanisms are far too slow to acutely regulate the GPL composition without energy wasting fluctuations (hysteresis). Those “coarse” mechanisms rather come into play when a change in GPL composition is required as, for example, during mitosis, cell differentiation or by altered cellular environment.^8^, ^43^, ^44^, ^45^ In principle, protein phosphorylation (or other modifications) could play a role in acute regulation of GPL compositions since those processes can take place rapidly and can influence protein activity.^46^, ^47^, ^48^, ^49^ However, this mechanism requires the existence of proteins that accurately “sense” the change in the GPL class composition of the membrane. As far as we are aware, no such proteins have been identified in mammalian cells so far. Notably, even if such sensor proteins do exist, they as well are likely to respond to variations in the chemical activity of the different GPL classes. In conclusion, abrupt variations in the chemical activity of the individual GPL classes are most probably the primary factor regulating GPL homeostasis.

## CONFLICT OF INTEREST

The authors made no disclosures.

## AUTHOR CONTRIBUTIONS

P. Somerharju, J. Virtanen, and M. Hermansson wrote the paper.

## References

[1] Hermansson M , Hokynar K , Somerharju P . Mechanisms of glycerophospholipid homeostasis in mammalian cells. Prog Lipid Res. 2011;50:240‐257. 21382416 10.1016/j.plipres.2011.02.004

[2] Sousa SB , Jenkins D , Chanudet E , et al. Gain‐of‐function mutations in the phosphatidylserine synthase 1 (PTDSS1) gene cause Lenz‐Majewski syndrome. Nat Genet. 2014;46:70‐76. 24241535 10.1038/ng.2829

[3] Sohn M , Ivanova P , Brown HA , et al. Lenz‐Majewski mutations in PTDSS1 affect phosphatidylinositol 4‐phosphate metabolism at ER‐PM and ER‐Golgi junctions. Proc Natl Acad Sci USA. 2016;113:4314‐4319. 27044099 10.1073/pnas.1525719113 PMC4843478

[4] Johansen A , Rosti RO , Musaev D , et al. Mutations in MBOAT7, encoding lysophosphatidylinositol acyltransferase I, lead to intellectual disability accompanied by epilepsy and autistic features. Am J Hum Genet. 2016;99:912916. 10.1016/j.ajhg.2016.07.019 PMC5065650 27616480

[5] Zhao T , Goedhart CM , Sam PN , et al. PISD is a mitochondrial disease gene causing skeletal dysplasia, cataracts, and white matter changes. Life Sci Alliance. 2019;2 10.26508/lsa.201900353. PMC6412922 30858161

[6] Wang B , Tontonoz P . Phospholipid remodeling in physiology and disease. Annu Rev Physiol. 2019;81:165‐188. 30379616 10.1146/annurev-physiol-020518-114444 PMC7008953

[7] Walkey CJ , Kalmar GB , Cornell RB . Overexpression of rat liver CTP:phosphocholine cytidylyltransferase accelerates phosphatidylcholine synthesis and degradation. J Biol Chem. 1994;269:5742‐5749. 8119913

[8] Jackowski S . Coordination of membrane phospholipid synthesis with the cell cycle. J Biol Chem. 1994;269:3858‐3867. 8106431

[9] Baburina I , Jackowski S . Cellular responses to excess phospholipid. J Biol Chem. 1999;274:9400‐9408. 10092620 10.1074/jbc.274.14.9400

[10] Barbour SE , Kapur A , Deal CL . Regulation of phosphatidylcholine homeostasis by calcium‐independent phospholipase A2. Biochim Biophys Acta. 1999;1439:77‐88. 10395967 10.1016/s1388-1981(99)00078-5

[11] Lykidis A , Wang J , Karim MA , Jackowski S . Overexpression of a mammalian ethanolamine‐specific kinase accelerates the CDP‐ethanolamine pathway. J Biol Chem. 2001;276:2174‐2179. 11044454 10.1074/jbc.M008794200

[12] Stone SJ , Cui Z , Vance JE . Cloning and expression of mouse liver phosphatidylserine synthase‐1 cDNA. Overexpression in rat hepatoma cells inhibits the CDP‐ethanolamine pathway for phosphatidylethanolamine biosynthesis. J Biol Chem. 1998;273:7293‐7302. 9516423 10.1074/jbc.273.13.7293

[13] Nishijima M , Kuge O , Maeda M , Nakano A , Akamatsu Y . Regulation of phosphatidylcholine metabolism in mammalian cells. Isolation and characterization of a Chinese hamster ovary cell pleiotropic mutant defective in both choline kinase and choline‐exchange reaction activities. J Biol Chem. 1984;259:7101‐7108. 6327706

[14] Polokoff MA , Wing DC , Raetz CR . Isolation of somatic cell mutants defective in the biosynthesis of phosphatidylethanolamine. J Biol Chem. 1981;256:7687‐7690. 6267019

[15] Fullerton MD , Hakimuddin F , Bonen A , Bakovic M . The development of a metabolic disease phenotype in CTP:phosphoethanolamine cytidylyltransferase‐deficient mice. J Biol Chem. 2009;284:25704‐25713. 19625253 10.1074/jbc.M109.023846 PMC2757972

[16] Fullerton MD , Bakovic M . Complementation of the metabolic defect in CTP:phosphoethanolamine cytidylyltransferase (Pcyt2)‐deficient primary hepatocytes. Metabolism. 2010;59:1691‐1700. 20427062 10.1016/j.metabol.2010.03.022

[17] Steenbergen R , Nanowski TS , Nelson R , Young SG , Vance JE . Phospholipid homeostasis in phosphatidylserine synthase‐2‐deficient mice. Biochim Biophys Acta. 2006;1761:313‐323. 16627002 10.1016/j.bbalip.2006.03.005

[18] Virtanen JA , Cheng KH , Somerharju P . Phospholipid composition of the mammalian red cell membrane can be rationalized by a superlattice model. Proc Natl Acad Sci USA. 1998;95:4964‐4969. 9560211 10.1073/pnas.95.9.4964 PMC20196

[19] Somerharju P , Virtanen JA , Cheng KH , Hermansson M . The superlattice model of lateral organization of membranes and its implications on membrane lipid homeostasis. Biochim Biophys Acta. 2009;1788:12‐23. 19007747 10.1016/j.bbamem.2008.10.004

[20] Batchu KC , Hokynar K , Jeltsch M , Mattonet K , Somerharju P . Substrate efflux propensity is the key determinant of Ca^2+^‐independent phospholipase A‐beta (iPLAbeta)‐mediated glycerophospholipid hydrolysis. J Biol Chem. 2015;290:10093‐10103. 25713085 10.1074/jbc.M115.642835 PMC4400325

[21] Hermansson M , Hanninen S , Hokynar K , Somerharju P . The PNPLA‐family phospholipases involved in glycerophospholipid homeostasis of HeLa cells. Biochim Biophys Acta. 2016;1861:1058‐1065. 27317427 10.1016/j.bbalip.2016.06.007

[22] Hermansson M . Homeostatic responses of cultured cell to phospholipid loading. Chem Phys Lipids. 2010;163:S23‐S24.

[23] Hermansson M , Hanninen S , Kjellberg MA , Somerharju P . (2019). A novel process maintaining glycerophospholipid homeostasis in mammalian cells. Bioachive. 10.1101/841221

[24] Kuge O , Nishijima M . Biosynthetic regulation and intracellular transport of phosphatidylserine in mammalian cells. J Biochem. 2003;133:397‐403. 12761285 10.1093/jb/mvg052

[25] Cornell RB , Ridgway ND . CTP:phosphocholine cytidylyltransferase: function, regulation, and structure of an amphitropic enzyme required for membrane biogenesis. Prog Lipid Res. 2015;59:147‐171. 26165797 10.1016/j.plipres.2015.07.001

[26] Arnold RS , Cornell RB . Lipid regulation of CTP: phosphocholine cytidylyltransferase: electrostatic, hydrophobic, and synergistic interactions of anionic phospholipids and diacylglycerol. Biochemistry. 1996;35:9917‐9924. 8703966 10.1021/bi960397c

[27] Dymond MK . Mammalian phospholipid homeostasis: homeoviscous adaptation deconstructed by lipidomic data driven modelling. Chem Phys Lipids. 2015;191:136‐146. 26375761 10.1016/j.chemphyslip.2015.09.003

[28] Imai A , Gershengorn MC . Regulation by phosphatidylinositol of rat pituitary plasma membrane and endoplasmic reticulum phosphatidylinositol synthase activities. A mechanism for activation of phosphoinositide resynthesis during cell stimulation. J Biol Chem. 1987;262:6457‐6459. 3032971

[29] Radhakrishnan A , Anderson TG , McConnell HM . Condensed complexes, rafts, and the chemical activity of cholesterol in membranes. Proc Natl Acad Sci USA. 2000;97:12422‐12427. 11050164 10.1073/pnas.220418097 PMC18778

[30] Sokolov A , Radhakrishnan A . Accessibility of cholesterol in endoplasmic reticulum membranes and activation of SREBP‐2 switch abruptly at a common cholesterol threshold. J Biol Chem. 2010;285:29480‐29490. 20573965 10.1074/jbc.M110.148254 PMC2937980

[31] Manguikian AD , Barbour SE . Cell cycle dependence of group VIA calcium‐independent phospholipase A2 activity. J Biol Chem. 2004;279:52881‐52892. 15385540 10.1074/jbc.M410659200

[32] Mancuso DJ , Sims HF , Han X , et al. Genetic ablation of calcium‐independent phospholipase A2gamma leads to alterations in mitochondrial lipid metabolism and function resulting in a deficient mitochondrial bioenergetic phenotype. J Biol Chem. 2007;282:34611‐34622. 17923475 10.1074/jbc.M707795200 PMC2980283

[33] Zaccheo O , Dinsdale D , Meacock PA , Glynn P . Neuropathy target esterase and its yeast homologue degrade phosphatidylcholine to glycerophosphocholine in living cells. J Biol Chem. 2004;279:24024‐24033. 15044461 10.1074/jbc.M400830200

[34] Bucher D , Hsu YH , Mouchlis VD , Dennis EA , McCammon JA . Insertion of the Ca(2)(+)independent phospholipase A(2) into a phospholipid bilayer via coarse‐grained and atomistic molecular dynamics simulations. PLoS Comput Biol. 2013;9:e1003156. 23935474 10.1371/journal.pcbi.1003156 PMC3723492

[35] McLean LR , Phillips MC . Kinetics of phosphatidylcholine and lysophosphatidylcholine exchange between unilamellar vesicles. Biochemistry. 1984;23:4624‐4630. 6498159 10.1021/bi00315a017

[36] Nichols JW . Thermodynamics and kinetics of phospholipid monomer‐vesicle interaction. Biochemistry. 1985;24:6390‐6398. 4084528 10.1021/bi00344a011

[37] Somerharju P . Is spontaneous translocation of polar lipids between cellular organelles negligible? Lipid Insights. 2015;8:87‐93. 27147824 10.4137/LPI.S31616 PMC4849424

[38] Huuskonen J , Olkkonen VM , Jauhiainen M , Metso J , Somerharju P , Ehnholm C . Acyl chain and headgroup specificity of human plasma phospholipid transfer protein. Biochim Biophys Acta. 1996;1303:207‐214. 8908155 10.1016/0005-2760(96)00103-8

[39] van Amerongen A , Demel RA , Westerman J , Wirtz KW . Transfer of cholesterol and oxysterol derivatives by the nonspecific lipid transfer protein (sterol carrier protein 2): a study on its mode of action. Biochim Biophys Acta. 1989;1004:36‐43. 2742872 10.1016/0005-2760(89)90209-9

[40] Balla T , Sengupta N , Kim YJ . Lipid synthesis and transport are coupled to regulate membrane lipid dynamics in the endoplasmic reticulum. Biochim Biophys Acta Mol Cell Biol Lipids; 2020; pii: S1388-1981(19)30075-7. 10.1016/j.bbalip.2019.05.005 PMC6858525 31108203

[41] Lange Y , Steck TL . Cholesterol homeostasis and the escape tendency (activity) of plasma membrane cholesterol. Prog Lipid Res. 2008;47:319‐332. 18423408 10.1016/j.plipres.2008.03.001 PMC2659507

[42] Lange Y , Ye J , Steck TL . How cholesterol homeostasis is regulated by plasma membrane cholesterol in excess of phospholipids. Proc Natl Acad Sci USA. 2004;101:11664‐11667. 15289597 10.1073/pnas.0404766101 PMC511035

[43] Sanchez‐Alvarez M , Zhang Q , Finger F , Wakelam MJ , Bakal C . Cell cycle progression is an essential regulatory component of phospholipid metabolism and membrane homeostasis. Open Biol. 2015;5:150093. 26333836 10.1098/rsob.150093 PMC4593667

[44] Murakami M , Kudo I , Inoue K . Change in phospholipid composition of mouse bone marrow‐derived mast cells during cultivation with fibroblasts. Biochim Biophys Acta. 1992;1124:17‐22. 1543721 10.1016/0005-2760(92)90120-k

[45] Sugimoto H , Banchio C , Vance DE . Transcriptional regulation of phosphatidylcholine biosynthesis. Prog Lipid Res. 2008;47:204‐220. 18295604 10.1016/j.plipres.2008.01.002

[46] Cardozo Gizzi AM , Caputto BL . Mechanistic insights into the nongenomic regulation of phospholipid synthesizing enzymes. IUBMB Life. 2013;65:584‐592. 23712998 10.1002/iub.1173

[47] Jacquemyn J , Cascalho A , Goodchild RE . The ins and outs of endoplasmic reticulumcontrolled lipid biosynthesis. EMBO Rep. 2017;18:1905‐1921. 29074503 10.15252/embr.201643426 PMC5666603

[48] Laplante M , Sabatini DM . An emerging role of mTOR in lipid biosynthesis. Curr Biol. 2009;19:R1046‐R1052. 19948145 10.1016/j.cub.2009.09.058 PMC3390254

[49] Guri Y , Colombi M , Dazert E , et al. mTORC2 promotes tumorigenesis via lipid synthesis. Cancer Cell. 2017;32: 807‐823.e12. 29232555 10.1016/j.ccell.2017.11.011

